# Monitoring Intracellular Metabolite Dynamics in *Saccharomyces cerevisiae* during Industrially Relevant Famine Stimuli

**DOI:** 10.3390/metabo12030263

**Published:** 2022-03-18

**Authors:** Steven Minden, Maria Aniolek, Christopher Sarkizi Shams Hajian, Attila Teleki, Tobias Zerrer, Frank Delvigne, Walter van Gulik, Amit Deshmukh, Henk Noorman, Ralf Takors

**Affiliations:** 1 Institute of Biochemical Engineering, University of Stuttgart, 70569 Stuttgart, Germany; steven.minden@ibvt.uni-stuttgart.de (S.M.); maria.aniolek@ibvt.uni-stuttgart.de (M.A.); c.sarkizi@ibvt.uni-stuttgart.de (C.S.S.H.); attila.teleki@ibvt.uni-stuttgart.de (A.T.); st163004@stud.uni-stuttgart.de (T.Z.); 2 Microbial Processes and Interactions (MiPI), TERRA Research and Teaching Centre, Gembloux Agro Bio Tech, University of Liege, 5030 Gembloux, Belgium; f.delvigne@uliege.be; 3 Department of Biotechnology, Delft University of Technology, van der Maasweg 6, 2629 HZ Delft, The Netherlands; w.m.vangulik@tudelft.nl; 4 Royal DSM, 2613 AX Delft, The Netherlands; amit.deshmukh@dsm.com (A.D.); henk.noorman@dsm.com (H.N.); 5 Department of Biotechnology, Delft University of Technology, 2628 CD Delft, The Netherlands

**Keywords:** scale-up, scale-down, metabolomics, bioreactor, systems biology, baker’s yeast, *Saccharomyces cerevisiae*, stimulus-response experiment, substrate gradient, bioprocess engineering, chemostat

## Abstract

Carbon limitation is a common feeding strategy in bioprocesses to enable an efficient microbiological conversion of a substrate to a product. However, industrial settings inherently promote mixing insufficiencies, creating zones of famine conditions. Cells frequently traveling through such regions repeatedly experience substrate shortages and respond individually but often with a deteriorated production performance. *A priori* knowledge of the expected strain performance would enable targeted strain, process, and bioreactor engineering for minimizing performance loss. Today, computational fluid dynamics (CFD) coupled to data-driven kinetic models are a promising route for the in silico investigation of the impact of the dynamic environment in the large-scale bioreactor on microbial performance. However, profound wet-lab datasets are needed to cover relevant perturbations on realistic time scales. As a pioneering study, we quantified intracellular metabolome dynamics of *Saccharomyces cerevisiae* following an industrially relevant famine perturbation. Stimulus-response experiments were operated as chemostats with an intermittent feed and high-frequency sampling. Our results reveal that even mild glucose gradients in the range of 100 μmol·L^−1^ impose significant perturbations in adapted and non-adapted yeast cells, altering energy and redox homeostasis. Apparently, yeast sacrifices catabolic reduction charges for the sake of anabolic persistence under acute carbon starvation conditions. After repeated exposure to famine conditions, adapted cells show 2.7% increased maintenance demands.

## 1. Introduction

Microbial catalysis has a pivotal role in realizing the transition from natural resource depletion towards a sustainable and circular economy [1,2]. Key factors underlining this status encompass the use of renewable feedstock, mild reaction conditions, a vast diversity of products and high potential for improving production efficiency and product quality—all benefitting from biological flexibility [3]. Consequentially, the European Horizon 2020 program recognizes biotechnology as one of four “Key Enabling Technologies” to maximize the sustainability and growth potential of European companies [4]. A prerequisite, but also one of the greatest challenges, is the successful transfer of lab results into commercial-scale bioreactors without the loss of performance [5,6,7,8]. This scale-up is often hampered by intrinsic drawbacks such as mixing insufficiencies, which, ultimately, cause a heterogeneous extracellular environment [9,10,11]. Numerous factors become increasingly dynamic, causing unexpected biological responses that either reduce the expected TRY (titer, rate and yield) criteria or even reveal a fatal potential for a given process [11,12].

To prevent the occurrence of detrimental scale-up effects, the inclusion of large-scale considerations into early-stage development is gaining more and more recognition in both the industry [13,14,15] and academic research [16,17,18,19]. Especially during substrate limited operation modes such as fed-batch or chemostat, concentration gradients can easily emerge, since volumetric reaction times are often within the same order of magnitude of the mean broth circulation times in an industrial environment [20,21]. Multiple investigations monitored cellular responses upon exposure to industrial conditions, aiming to explain the observed performance losses. Industrial hosts were exposed to substrate heterogeneities, revealing an overflow metabolism [22,23], the disturbance of energy management [24,25] and perturbations of regulatory programs mirrored by metabolomics [26,27], transcriptomics [28,29] and proteomics [30,31,32]. Even a population heterogeneity was observed [33,34].

**Figure 1:**
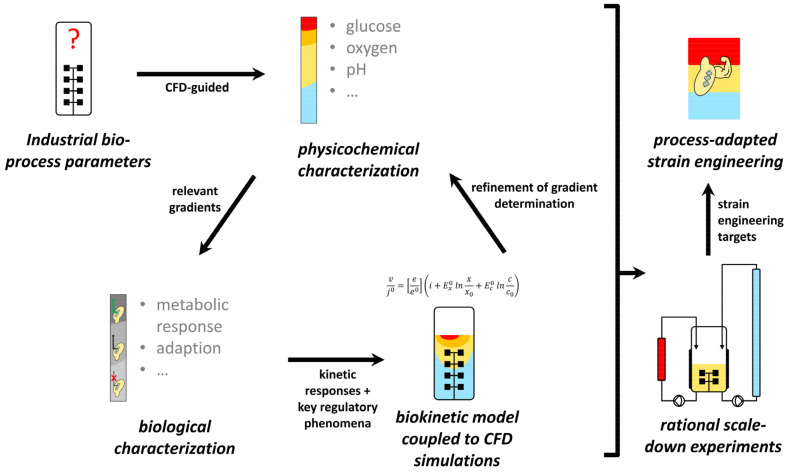
Basic procedure for data-driven scale-up/scale-down development. Concentration gradients are derived from large-scale simulations to design stimulus-response experiments and generate *-omics* datasets. This approach further allows the set-up of biological models to refine large-scale simulations. Ultimately, gained knowledge enables process-adapted strain engineering and the design of realistic scale-down simulators for validation experiments to replace classical scaling-up.

How can scale-down experiments be designed to adequately reflect industrial hydrodynamics and reaction dynamics when large-scale data are usually not available? Modern bioprocess development strategies substitute this knowledge gap with simulations based on computational fluid dynamics (CFD) coupled to biokinetic models [6,21,35]. This setup allows integrating exchange rates with the hydrodynamic environment of the bioreactor. More precisely, the exposure of individual microorganisms to substrate gradients can be recorded during all process phases and expressed as lifelines [36]. Currently, this approach reaches considerable agreement with quantitative data on concentration gradients from pilot to industrial scale [37,38,39,40]. An adjacent development goal is to increase the predictive power to uncover biological scale-up effects already at the development stage in the lab via data-driven models. Thus, comprehensive *-omics* data for model development are paramount and can, for instance, be provided by stimulus-response experiments (SRE) that capture relevant large-scale dynamics. Figure 1 demonstrates a conceptual workflow with integrated wet- and dry-lab contributions. Ultimately, the generated knowledge allows both the identification of strain engineering targets and the quantitative design of scale-down simulators to replace physical upscaling. A successful archetype for this strategy has recently resulted in the construction of an *E. coli* strain with reduced maintenance energy demands when subjected to industrial glucose gradients [29,41].

This work is part of a case study with the ambition to deploy the aforementioned rational bioprocess engineering approach for a eukaryotic model organism. *Saccharomyces cerevisiae* was chosen due to its broad prevalence in several sectors of the bioprocessing industry, comprising foods, fuels, chemicals and pharmaceuticals [42,43]. The industrial setting is derived from a 22 m^3^ research bioreactor operated as a glucose-limited fed-batch process for biomass production, which is thoroughly described in the literature [37,44,45]. Corresponding CFD investigations and large-scale measurements already identified glucose gradients in the range of 23–460 μmol·L^−1^ [22,38,39]. This distinct concentration spectrum favors the emergence of three metabolic regimes: First, the desired operating point in the glucose-limited state to achieve an optimal biomass conversion. Second, overflow metabolism due to a glucose excess close to the feeding position. Third, starvation regimes far away from the feed, where the glucose uptake cannot satisfy cellular maintenance demands anymore.

The before-mentioned SRE approach represents a proven methodology to provide the necessary ground to set up data-driven models [46,47,48]. For the organism under investigation, several studies quantitatively investigated the metabolome and transcriptome during a sudden shift from a glucose limitation to excess [26,47,49,50]. To the best of our knowledge, the current state of the literature is missing complementary data for the opposing transition between limitation and starvation. This study, therefore, set out to close this gap of knowledge, beginning on the metabolic level. On the one hand, quantitative endometabolomic measurements provide a sound database for a more detailed model development. On the other hand, the interpretation of the dataset uncovers biological mechanisms that can lead to strain performance losses for different production scenarios and guide large-scale adapted strain engineering.

## 2. Results

### 2.1. Hyperbolic Kinetics Overestimate Starvation Regimes in Industrial-Scale Simulations

**Figure 2:**
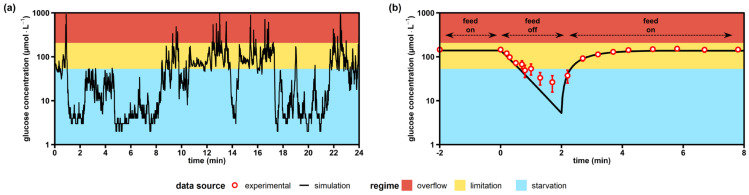
Simulated versus experimental glucose profiles experienced by yeast cells. (**a**) Exemplary lifeline of a single *Saccharomyces cerevisiae* trajectory recorded over 24 min during an industrial glucose-limited fed-batch process with a biomass concentration of 10 g·L^−1^. The lifeline was simulated during the work of Sarkizi et al. [39], but not published. (**b**) Stimulus-response experiment as a glucose-limited chemostat with intermittent feed (this work). Extracellular glucose levels are the means ± standard deviation of six biological replicates (merged trends from adapted and non-adapted time series). All simulated values were computed using published glucose uptake kinetics [51]. Overflow metabolism was assumed to start at glucose concentrations >207 μmol·L^−1^ [52] and starvation zones developed below 53 μmol·L^−1^, where maintenance demands could not be covered anymore [53].

Figure 2a presents 24 min of a three-hour single-cell lifeline mimicking the late stage of an industrial baker’s yeast production scenario. The simulation suggests that cells resided only 39% of the time in the favored glucose limitation regime, delivering the planned substrate supply for growth and maintenance. Moreover, overflow regimes occurred, lasting for 1–10 s and making up 3% of the lifeline. However, for 58% of the lifeline, the yeast trajectory was subject to severe starvation conditions, which caused the famine status to be the normality rather than the exception.

To mimic the dominant role of glucose starvation, we exposed yeast cells to famine conditions (Figure 2b). In the glucose depletion experiment, minimal glucose levels of 22 μmol·L^−1^ were found after the feed was stopped for 2 min. Interestingly, simulations using the kinetic parameters of Figure 2a predicted residual glucose levels well below 10 μmol·L^−1^. However, the semilogarithmic slope of the experimental limitation-starvation transition in Figure 2b was only 0.44 s^−1^, which accounted for 60% of the anticipated kinetics (0.71 s^−1^). Apparently, additional impacts occurred that hampered the one-by-one application of the stated hyperbolic uptake kinetic for the short-term starvation.

Nevertheless, it was concluded that cellular exposure to famine conditions was a dominating scenario in large-scale bioreactors. Accordingly, follow-up studies considered 2 min starvation intervals that allowed the investigation of endo-metabolite dynamics for two scenarios: (i) a single limitation–starvation–limitation (LSL) cycle revealing the nonadapted cellular response and (ii) a representative LSL cycle from an adapted culture.

### 2.2. Process and Phenotypic Characterization

The haploid *S. cerevisiae* strain CEN.PK 113-7D was cultivated in glucose-limited, aerobic chemostats in biological triplicates, each carried out with a dilution rate of 0.1 h^−1^. Three experimental phases were investigated: (i) The first period operated stably for five residence times serving as the reference steady state (RS). (ii) Then, the feed was inactivated once for 120 s to install starvation conditions. Subsequently, previous feeds were re-installed and the post-starvation response was tracked for 360 min. (iii) During the third phase, a periodic feed regimen with cycles of 2 min starvation and 7 min limitation was implemented, operating for five residence times to establish a new steady state after dynamic stimuli (DS).

Table 1 lists recoveries of carbon, nitrogen and available electrons (*ave*) for steady-state RS, samples after the first LSL cycle and for steady-state DS. Notably, all balances closed within 100 ± 5%. Except for minor amounts of trehalose and glycerol (data not shown), no by-product formation was detected, which agreed with similar studies using CEN.PK 113 7D [54,55]. Only acetic acid formation was reported under reference conditions [55], which did not occur in our study. The carbon balance of the ‘30 min post-stimulus’ sample was the only significant deviation from the reference steady state (*p*-value < 0.05). In this phase, respiratory dynamics (see Section 2.3) estimated by the mathematical off-gas deconvolution method might have caused a measurement error, since both the CO_2_-dependent carbon and O_2_-dependent *ave* recoveries were affected by the same increase.

The phenotypic characterizations of the steady states RS and DS are presented in Table 2. Prominent differences were observed for biomass-specific oxygen demands and carbon dioxide emissions in DS, each rising by 4.3%. Although *Y_DMB_*_/glucose_ and the glucose uptake rate (*q*_glucose_) remained unchanged in RS and DS, changes in the oxygen uptake and carbon dioxide release pointed towards metabolic rearrangements. Furthermore, the adapted cells of DS appeared to possess a superior cellular integrity, since the leakage of unknown carbon was reduced by 13.4%, which is an indicator for cell lysis [56].

Summarizing, the comparison of steady-state phenotypes hinted to elevated ATP needs at DS that were mirrored by an increased oxygen uptake and carbon dioxide formation rates. Consequently, time-resolved studies were performed to uncover underlying mechanisms.

### 2.3. Short-Term Metabolome Relaxation Requires 7 min after Glucose Repletion

**Figure 3:**
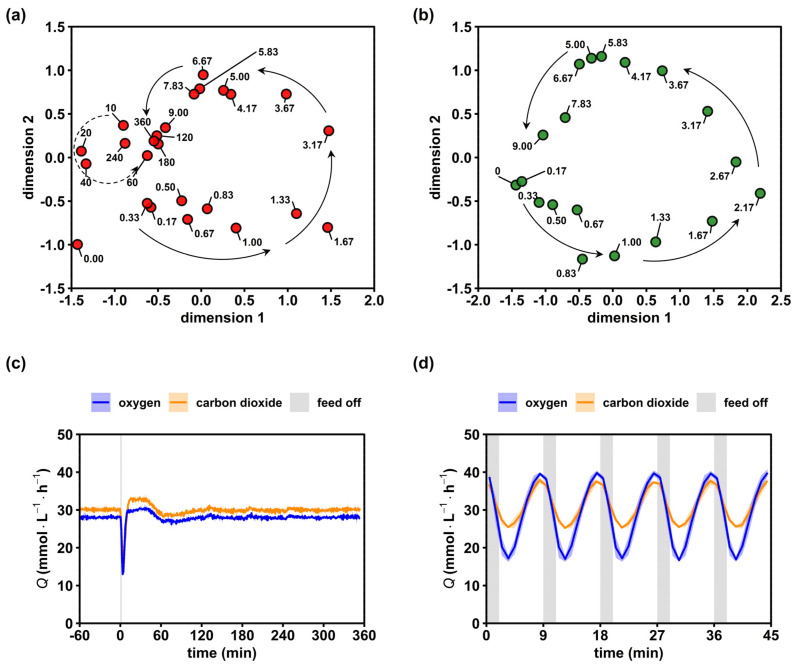
Relaxation of the intracellular metabolome and respiratory activity. (**a**) Multidimensional scaling (MDS) plot of the non-adapted (red) 6 h time series based on min–max normalized concentrations of 28 intracellular metabolites. Arrows provide a visual aid to follow the short-term (solid) and mid-term (dashed) dynamics. (**b**) Analogous MDS plot of the adapted (green) 9 min time series. (**c**) Evolutions of the oxygen and carbon dioxide transfer rates after a single starvation transition. (**d**) Analogous off gas analysis over 5 perturbation cycles during the dynamic steady state. Text labels in (**a**,**b**) represent the sample time in minutes. Blue and orange lines in (**c**,**d**) represent the mean and light areas represent the respective standard deviation of three biological replicates.

Metric multidimensional scaling (MDS) plots of the quantified intracellular metabolome and respiratory activity were used as proxy variables to visualize the relaxation pattern of intracellular dynamics in non-adapted and adapted cells. By trend, Figure 3a resembles a spiral-type trajectory of metabolite levels converging to the ‘9 min’ spot. Remarkably, late time points 240 and 360 min did not approximate the reference steady state (0.00 min). This result was rather unexpected, since the maximum turnover times for the reported metabolites were in the range of 1 × 10^0^–1 × 10^2^ s [57] and, thus, two orders of magnitude shorter than the observed time window. Instead, the observation may be taken as a hint on the flexibility of the metabolome, enabling similar growth phenotypes with different compositions of intracellular metabolite patterns. Further evidence was provided later. Maybe even more surprising was the continuing phenotype dynamics of the oxygen uptake and CO_2_ formation during 10–60 min (Figure 3c), although the metabolome seemed to have already relaxed after converging to the ‘attractor’ point of the 9 min sample. Together, these results unraveled the existence of a first, immediate response to a glucose shortage lasting for about 9 min, and a second, less pronounced dynamic between 10 and 60 min.

For investigating the adapted response, the final metabolite cycle (Figure 3b) after multiple stimulations was expressed in the MDS space. Other than the non-adapted response, we observed no spiral but rather a circular 9 min trajectory without a distinct convergence. This reflected the dynamics in the off-gas analysis (Figure 3d), showing highly repeatable amplitudes of *Q*_oxygen_ and *Q*_carbon dioxide_ with 22.8 ± 0.3 mmol·L^−1^·h^−1^ and 12.3 ± 0.2 mmol·L^−1^·h^−1^, respectively. Notably, off-gas dynamics were always observed in biological triplicates lasting for more than 10 cycles (only 5 were shown). The high reproducibility of the phenotype gave rise to the assumption that the metabolite cycles of Figure 3b equally repeated in the perturbation series.

Summarizing, results indicated that an observation window of nine minutes covered the first, immediate cellular response on the glucose shortage. Differences between the adapted and non-adapted cell response existed that may have been elucidated by the analysis of intracellular metabolite dynamics.

### 2.4. Short-Term Dynamics of the Central Catabolism upon Glucose Depletion

**Figure 4:**
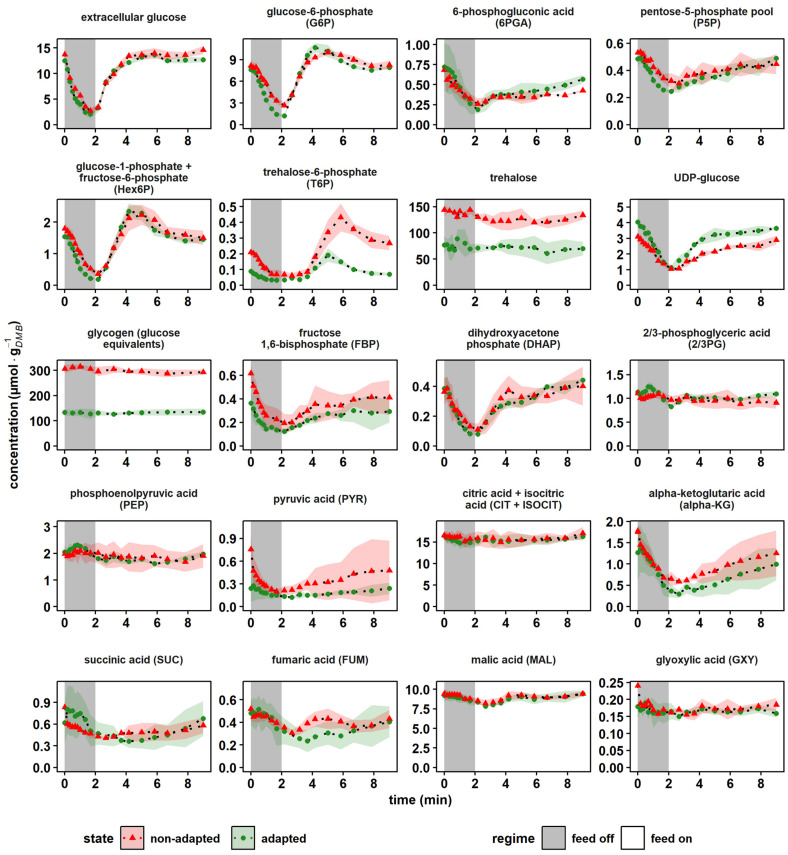
Dynamics of central catabolic metabolites after a 2 min glucose depletion phase. The nonadapted response (red) indicates dynamics following a single transition into a starvation scenario (“feed off” phase) and the adapted response (green) was sampled from representative 9 min cycles during steady-state DS. Time point 0 min of the non-adapted response was equal to steady-state RS. All values indicate means ± standard deviation of three biological replicates.

To elucidate the phenotypic differences shown by non-adapted and adapted cells after exposure to a glucose limitation, we investigated the time course of selected intracellular metabolites involved in the glucose catabolism (Figure 4).

The central upper glycolysis metabolites glucose-6-phosphate (G6P) and the merged glucose-1-phosphate/fructose-6-phosphate pool (Hex6P) both qualitatively followed the extracellular availability of glucose, irrespective of the cellular adaption status. However, a slight overshooting of about 29% occurred in adapted cells (green) for the minimum and maximum G6P compared to the extracellular glucose amplitudes (*p*-value < 0.05). The hexokinase reaction was feedback-inhibited by trehalose-6-phosphate (T6P) [58,59] and indeed, on average, the T6P pool decreased by 52% in the adapted yeast population, possibly resulting in reduced control over the hexokinase activity. Furthermore, a sharp rise of T6P coincided with peaking G6P levels. Apparently, large G6P pools triggered the carbon drain into the storage compound trehalose via T6P. Interestingly, the total levels of the carbon storage buffers trehalose and glycogen were reduced in adapted versus nonadapted cells by 43% and 49%, respectively. As these pool sizes are reported to correlate inversely with the growth rate [60,61], which was kept constant in the experimental series, the finding was unexpected. Assuming a carbon ratio of 0.04 mol_C_·g*_DMB_*^−1^ [62], the reduction in the carbohydrate pools should account for a 4% drop in *Y_DMB_*_/glucose_. Because the latter was not observed (Table 2), we assumed that the substantial metabolic re-arrangement should have occurred in adapted cells. Further hints were provided by the elevated average concentrations of UDP-glucose (+31%) in adapted cells. UDP-glucose not only links glycolysis with the carbohydrate storage pools, but plays a key role in the anabolism of structural components such as cellulose, β-glucan, glycolipids and glycoproteins [63]. Consequently, increased UDP-glucose levels may reflect the observed increase in the cellular integrity (Table 2) of adapted cells.

**Figure 5:**
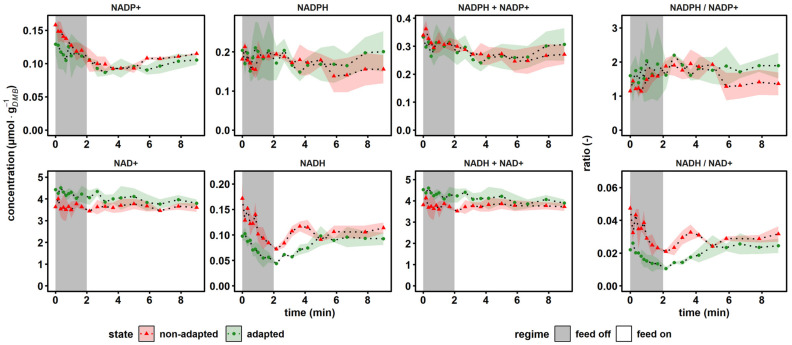
Dynamics of the reduction equivalents, conserved moieties and according to ratios. The non-adapted response (red) indicates dynamics following a single transition into a starvation scenario (“feed off” phase) and the adapted response (green) was sampled from representative 9 min cycles during steady-state RS. Time point 0 min of the non-adapted response was equal to steady-state RS. All values indicate means ± standard deviation of three biological replicates (except for the non-adapted time series, which was derived from two biological replicates).

**Figure 6:**
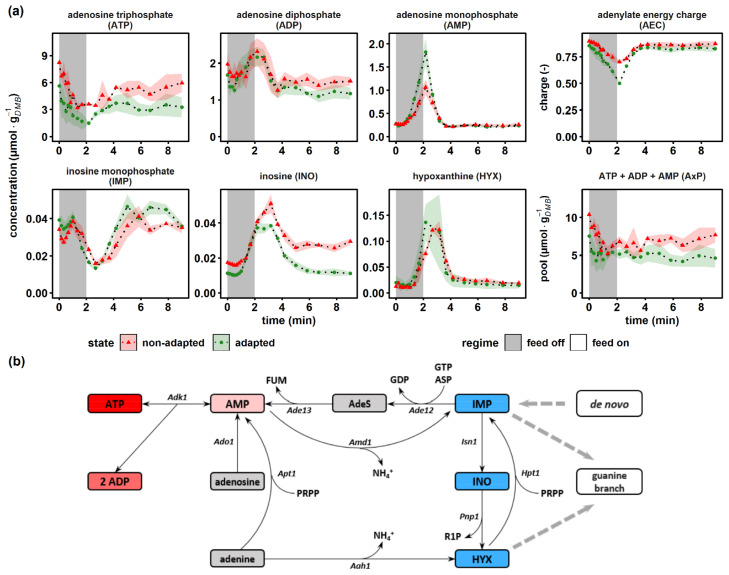
Dynamics of energy carriers and intermediates of the purine salvage pathway. (**a**) The non-adapted response (red) indicates dynamics following a single transition into a starvation scenario (“feed off” phase) and the adapted response (green) was sampled from representative 9 min cycles during steady-state DS. Time point 0 min of the non-adapted response was equal to steady-state RS. The adenylate energy charge was calculated according to [72]. All values indicate means ± standard deviation of three biological replicates. (**b**) Schematic representation of the adenylate kinase system attached to the purine salvage pathway, reproduced from [73,74]. *Aah1*, adenine deaminase; *Ade12*, adenylosuccinate synthase; *Ade13*, adenylosuccinate lyase; AdeS, adenylosuccinate; *Adk1*, adenylate kinase; *Ado1*, adenosine kinase; *Amd1*, AMP deaminase; *Apt1*, adenine phosphoribosyl transferase; ASP, aspartate; FUM, fumarate; *Hpt1*, hypoxanthine-guanine phosphoribosyl transferase; *Isn1*, IMP-specific 50-nucleotidase; *Pnp1*, purine nucleoside phosphorylase; PRPP, phosphoribosyl pyrophosphate; R1P, ribose-1-phosphate.

Regarding the short-term dynamics of the intermediates of the pentose phosphate pathway (PPP), two phases could be observed. Interestingly, they were similar for adapted and non-adapted cells. During the first 2 min of nascent glucose depletion, the trends of 6-phosphogluconic acid (6PGA) and the merged pool of ribose-5-phosphate and ribulose-5-phosphate (P5P) followed the extracellular glucose availability. Then, the recovery to initial pool sizes was delayed and somewhat disconnected from the external glucose supply. The observation agreed with findings of Theobald et al. and Suarez-Mendez et al., who applied glucose pulse experiments [49,50]. They hypothesized a dominating glycolytic flux control over PPP, a conclusion that was complemented by additional cofactor and sink reaction measurements presented and discussed in Figure 5 and Figure 6a.

Similar trends of delayed recovery were also observed for fructose-1,6-bisphosphate (FBP). Phosphofructokinase (PFK) delivering FBP is well known to be inhibited by ATP and citrate (CIT) and activated by ADP, AMP, F6P and fructose-2,6-bisphosphate (F2,6BP, not quantified) [64,65]. Noteworthy are the in vitro and in vivo studies by van den Brink et al., revealing that metabolic regulations of PFK may be superimposed by upshifting glycolytic fluxes if energy homeostasis is impaired [66]. The latter likely occurred during the first 2 min of the experiments (see Figure 6a).

Further down in glycolysis, pools of 2- and 3-phosphoglycerate (2/3PG) and phosphoenolpyruvic acid (PEP) showed surprisingly few perturbations irrespective of whether non-adapted or adapted cells were studied. Either related metabolite consumption completely stopped or compensating fluxes occurred. Given the fast turnover rates of the stated pools typically ranged in seconds, the latter is the likely explanation. Further considering that trehalose and glycogen pool sizes persisted even during the first 2 min of nascent starvation, the start of gluconeogenesis is a plausible scenario. Pyruvate kinase (PKY) converting PEP + ADP into PYR + ATP is well known to be activated by FBP, which, interestingly enough, dropped severely by 61% from 0.36 ± 0.10 μmol·g*_DMB_*^−1^ to 0.14 ± 0.02 μmol·g*_DMB_*^−1^. Because of the missing downwards flux, gluconeogenesis was induced [67], causing stable upstream pool sizes.

In the tricarboxylic acid cycle (TCA), intermediates showed similar trends in all conditions. The merged pool of citric acid (CIT) and isocitric acid (ISOCIT) kept constant, whereas the downstream intermediate α-ketoglutaric acid (αKG) mirrored the extracellular glucose shortness of the first 2 min, followed by a delayed recovery. This trend was visible in all subsequent TCA metabolites, although dampened with an increasing reaction distance to αKG. This finding was in agreement with earlier studies of Mashego et al., who performed glucose pulse experiments, observing stronger perturbation dynamics for αKG than for CIT [47]. Presumably, the trends reflect the mitochondrial export of αKG into the cytosol for oxidative nitrogen fixation in glutamic acid [68]. Unfortunately, no dynamic glutamic acid measurements were available in this study.

Taken together, LSL perturbations were propagated on separating time scales through the central metabolic nodes of *S. cerevisiae*. Moreover, the adaption status was most visible in the pool sizes of carbon storage buffers.

### 2.5. Analysis of Anabolic and Catabolic Reduction Equivalents

The dynamics of the nicotinamide electron carriers are depicted in Figure 5. The upper panel indicates individual concentrations of the anabolic redox pair NADP^+^, NADPH, their sum and their ratio. By analogy, the catabolic redox state is indicated in the second row.

Regarding anabolic reduction, the total pool size of 0.36 ± 0.00 μmol·g*_DMB_*^−1^ and the reductive ratio of 1.28 ± 0.01 measured at reference conditions agreed with literature values for CEN.PK113-7D, which were observed in glucose-limited chemostat at *D* = 0.1 h^−1^ as 0.25–2.17 μmol·g*_DMB_*^−1^ and 0.29–4.86, respectively [69]. By trend, NADP^+^ pool sizes dropped during glucose depletion, both for adapted and non-adapted cells, which led to rising anabolic reduction charges.

In contrast, NAD^+^ concentrations remained virtually unchanged during glucose depletion, whereas NADH levels decreased. Interestingly, in the non-adapted scenario, the recovery of the NADH pool was not observed within the 9 min time window, but in the adapted case, full relaxation was reached after 5 min. However, NADH levelled out at 0.10 ± 0.01 μmol·g*_DMB_*^−1^, which was 43% less than the reference state at 0.17 ± 0.03 μmol·g*_DMB_*^−1^. The lumped pool size remained stable during the perturbation, since only NADH showed dynamics, which only accounted for approximately 4% of the total pool size. Literature values for the catabolic reduction charge under comparable steady-state conditions ranged from 0.05 to 0.2 [50,70,71], which was somewhat larger than the reference value of 0.046 ± 0.009 measured for the non-adapted yeast. The observation mirrored the twofold increased NAD^+^ concentrations of this study work versus the respective levels in the cited studies that yielded ratios above 0.1.

Summarizing, the results indicated opposite trends during nascent glucose starvation: while the anabolic reduction state rose, the catabolic dropped. Or, in other words, NADPH and NAD^+^ levels persisted, whereas NADP^+^ and NADH pool sizes dropped.

### 2.6. The Adenylate Energy Charge Is Quickly Regenerated at the Cost of Total Adenylate Pool Size

Energy carrier homeostasis and nucleotide resource management during dynamic glucose availability were monitored via adenylate and selected purine salvage pathway (PSP) intermediates (Figure 6a). The adenylate energy charge (*AEC*) was calculated based on the original approach from Atkinson et al. [72]. The ATP concentration decreased from 8.12 ± 0.72 μmol·g*_DMB_*^−1^ to 3.56 ± 0.16 μmol·g*_DMB_*^-1^ and from 5.63 ± 1.54 μmol·g*_DMB_*^−1^ to 1.60 ± 0.61 μmol·g*_DMB_*^−1^ within 120 s in the non-adapted and adapted scenarios, respectively. In the same interval, AMP displayed a sharp 3.9-fold (non-adapted) and 6.6-fold (adapted) peak, while ADP first dropped before rising after 30 s with a maximum coinciding with that of AMP. Adenylate energy charges of non-adapted and adapted yeasts showed physiological values of about 0.90 ± 0.03, which dropped during glucose starvation before recovering again to the initial value. Interestingly, the drop of *AEC* was more pronounced in adapted cells. However, both cells had in common that total AxP pools reduced during glucose starvation and did not fully replenish during the post-starvation period. Apparently, physiological *AEC* values of about 0.9 observed after famine exposure were achieved at the cost of ADP pools that did not recover to the prestarvation values.

Remarkably, the similar phenotype of the *AEC* adjustment at the cost of AxP reduction was reported in glucose pulse studies [26,46,47,73]. Kresnowati et al. and Walther et al. hypothesized that nucleotide salvage mechanisms may explain the underlying mechanism of the observation. Adenine nucleotides were shuttled into the PSP via the AMP deaminase (AMD1) reaction yielding inosine monophosphate (IMP), the central intermediate for both, de novo and salvage pathways of purines (Figure 6b). At this branch point, IMP could either (i) enter a futile cycle where AMP is regenerated at the expense of GTP and aspartate, yielding GDP and fumarate; (ii) be interconverted via inosine (INO) to hypoxanthine (HYX) back to IMP at the expense of ribose-1-phosphate (R1P) and phosphoribosyl pyrophosphate (PRPP) or (iii) be shuttled towards the guanine salvage branch catalyzed by the NAD^+^-dependent IMP dehydrogenase (IMD2,3,4) [73]. Surprisingly, the pattern of IMP under famine conditions resembled the oscillatory behavior of ADP rather than that of the IMP precursor AMP. The IMP levels displayed a second decline phase after 1 min, coinciding with strongly increasing inosine and hypoxanthine levels. The latter accumulated to their maximum concentrations about 1 min later than AMP, their common upstream intermediate. Interestingly, INO pools of non-adapted cells remained 1.6-fold elevated compared to the prestarvation condition. This may be interpreted as a ‘memory’ effect that was not shown by adapted cells.

## 3. Discussion

### 3.1. Decreased Glucose Uptake Kinetics

Several reports have shown that a variable substrate availability is a fundamental scale-up effect causing observed strain performance losses in industrial fed-batch processes [22,23,45,75]. The investigated case of glucose limited *S. cerevisiae* CEN.PK113-7D exemplified the cellular responses at μ = 0.1 h^−1^ when the substrate concentration c_S_ was 10-fold lower than the affinity constant K_M_ of the most efficient hexose transporters HXT6 and HXT7 [51,76]. If c_S_ << K_M_, the glucose uptake kinetics are proportional to extracellular concentrations [77], which may explain the observed deviation between predictions based on hyperbolic kinetics and the experimental observation in our study (Figure 2) and in other works [50]. This discrepancy could be attributed to the presence of a secondary source of extracellular glucose in the form of exported trehalose. The disaccharide is hydrolyzed in the extracellular space by the free acid trehalase Ath1, which has an optimum at the operated pH of 5.0 [78]. Comparable fermentation studies investigating ^13^C labeling patterns traced the presence of unlabeled glucose to trehalose breakdown. Furthermore, there are several other theoretical indications to consider, such as the decoupled glucose uptake and sensing [79] or the inhibition of the glucose uptake by intracellular glucose [80] concomitant with glucose secretion due to the reversibility of facilitated diffusion [81]. Thus, several aspects of glucose transport and even additional source reactions must be considered for optimal glucose characterization at the boundary of starvation, as they can play an important role in computing realistic large-scale simulations.

### 3.2. Exposure to Starvation Revealed Different Tactics of Reserve Management

Macroscopic observations indicated the emergence of a new growth phenotype of adapted cells compared to non-adapted cells. The first managed to maintain the same biomass/substrate yield, while the respiratory activity rose and carbon storage pools remained on a lower, but constant, level. Given that carbon dioxide emission rates of adapted cells increased by about 4.3% while the glucose uptake rates kept constant, one may anticipate a likewise dip of *Y_DMB_*_/glucose_ that did not occur. Therefore, the cells should have found alternative resource allocation possibilities targeting proteins. The hypothesis was consistent with strongly reduced amino acid pools (Table A2) and an observed 9% increase in the ammonia uptake rate during the dynamic steady state (Table 2). In general, the rearrangement of the cellular composition is a fundamental strategy of *S. cerevisiae* to adjust to new environmental conditions through balancing growth against maintenance [82]. Fast growth, for instance, is accompanied by high ribosomal contents tapping into storage carbohydrates to ensure anabolic needs [83,84]. A similar cellular strategy was revealed in the current study, most likely to support the increased maintenance demands rather than to elevate growth. Indeed, estimating *q*_ATP_ assuming a P/O ratio of 1.08 yielded a significant 2.7% increased ATP demand in adapted cells [85].

Considering intracellular metabolite pool sizes, differences between the adapted and non-adapted states mirrored the adaptation of the yeast to cultivation conditions. Prolonged carbon-limited chemostat cultivations by Mashego and Jansen et al. [86,87] already revealed decreasing pool sizes of maximum 20% after 10 generations that were interpreted as the consequence of selection pressure. The present study also encompassed approximately 10 generations between steady states RS and DS. Consequently, minor pool size reductions <20% should be ignored to separate the effects of long-term growth selection from the results of metabolic rearrangement because of the dynamic stimuli. Still, key findings outlined above should be valid. For instance, trehalose and glycogen pool reductions were likely to be a consequence of the repeated exposure to famine conditions. This made sense from an economic point of view, given the relatively high contribution of both pools towards ATP dissipation via futile cycling [55]. Other evidence towards a more energy-saving mode in adapted cells was derived from the twofold increased AMP peak compared to the non-adapted yeast. High AMP levels activate the PFK enzyme while simultaneously inhibiting the reverse reaction catalyzed by FBP and, consequentially, further reduce ATP dissipation in the F6P–FBP futile cycle [88]. Hence, during adaption, the non-growth-associated ATP usage appeared to be increased and rebalanced for supporting other maintenance components than energy buffering.

There remains the question of which relationship elicited the emerging new phenotype when the same net rates of growth and substrate uptake prevailed. As mentioned earlier, glucose uptake and sensing are decoupled processes in *S. cerevisiae* [79]. Zaman et al. characterized the transcriptional response of conditional mutants against different glucose sensing scenarios. The authors concluded that extracellular glucose sensing could indeed induce strong phenotypic changes, while the same net influx of glucose prevails [89]. Whether the decoupled substrate uptake and sensing explain the present observation should be addressed in future research to fully understand the regulatory mechanisms that shape the industrial phenotype.

### 3.3. The Cellular Strategy to Ensure Anabolic Demands

The concentration profiles of most intermediates of the upper glycolysis and tightly linked metabolites followed the decline in extracellular glucose levels. However, during the transition from starvation back to the new steady state, time scales of pool relaxation were partially decoupled from glucose availability. The differences of recovery dynamics reflected different flux patterns that apparently mimicked cellular needs. For instance, the PPP reached pre-perturbation levels 4 min later than its precursor G6P. Considering that steady-state glycolytic flux was about 20-fold larger than the branching flux into PPP [55], its pools needed longer to recover. Apparently, this reflected the cellular program to prioritize catabolic over anabolic activity. Saliola and colleagues reported that most eukaryotic G6P dehydrogenases (*Zwf1* in *S. cerevisiae*) possess both a catalytic binding site for NADP^+^ and an allosteric binding site for NADPH [90]. This allows the cell to drain fluxes towards glycolytic catabolism, thereby gaining ATP either via *Zwf1* inhibition under NADPH excess or via NADP^+^ limitation. Apparently, the second occurred during the SRE experiments.

Interestingly, neither trehalose nor glycogen pools were degraded during the short-term exposure to glucose starvation. This was in line with previous observations, where short-term glucose perturbations on the same time scale did not change glycogen [27,91] or trehalose concentrations [27], even though rapid trehalose mobilization is anticipated in the literature [92]. This disagreement may be explained as follows: cytosolic trehalase is dependent on activation via a cAMP-dependent prost-translational modification (PTM) cascade yielding its phosphorylation [92]. However, the adenylate cyclase *Cyr1* in CEN.PK113-7D carries a mutation that causes a delay in trehalose and glycogen mobilization [93]. Consequentially, the short-term persistence of glycogen and trehalose pools may be a distinct feature of the current strain, and may be different in other genotypes that were not selected after growth evolution.

Intracellular metabolite dynamics were less pronounced in lower glycolysis and in TCA. In some cases, a high variance additionally hindered a statistically sound interpretation (e.g., for PYR). However, the quick reduction in the catabolic reduction charge under famine conditions might be the consequence of a reduced flux into the TCA, since the onset of gluconeogenesis was observed. In essence, reactions generating NADH, such as oxoglutarate decarboxylase (OGDC), isocitrate (IDH) and malate dehydrogenases (MDH), were reduced. With the missing influx, pools of αKG reduced quickly, indicating that the drain into amino acid synthesis and the production of glutamate remained. Notably, αKG may be regarded as an alarmone, being at the intersection of oxidative carbon and nitrogen metabolism. The reductive amination to form glutamate is tightly controlled by the redox status of the NADP^+^/NADPH couple [94]. Considering the rising NADPH/NADP^+^ ratio (Figure 5), glutamate formation was likely to continue even during famine conditions. Together with the observation of falling NADH/NAD^+^ ratios, the conclusion could be drawn that the yeast favored anabolism for the sake of catabolism under short-term carbon starvation.

Ultimately, decreasing catabolic reduction power impaired energy homeostasis due to an imbalance in the electron transport chain. With reducing glycolytic fluxes, ATP gain via respiration became even more important under famine conditions. Consequently, the falling NADH supply was proportionally reflected in likewise falling ATP levels. The increasing ADP:ATP ratio pushed the adenylate kinase 1 (*Adk1*) away from its equilibrium to catalyze the conversion of ADP to ATP and AMP [95]. This correlation might also explain the larger AMP peak in adapted cells, since the ADP:ATP ratio increased by approximately 15% compared to non-adapted cells. AMP accumulation was prevented via removal towards INO via IMP using the purine salvage pathway. As no obvious regulatory roles could be assigned to IMP and INO thus far, Walther and colleagues suggested that AMP is shuttled to PSP to reduce its regulatory impact, which may partially explain the delayed regeneration of the AxP pool after stress relief [73].

### 3.4. Consequences for Production Scenarios with Saccharomyces cerevisiae

Dynamic environments in industrial-scale bioreactors can induce manifold cellular responses. Carbon-limited fed-batch processes typically operate at carefully designed substrate supply optima, which could be easily inferred once cells enter zones of substrate depletion. The latter often occur far away from the feed inlet [38] or in areas with poor mixing. The current study identified a number of intracellular responses that have the potential to impair the yeast performance in large-scale production scenarios. For instance, dynamic extracellular LSL transitions caused the emergence of a new phenotype with possible implications in recombinant protein production. Increased maintenance demands could directly compete with energetic demands for protein production in the form of an added metabolic burden [96]. Another point to consider might be the failure of cellular buffering capacities to counterbalance rapid substrate perturbations. For instance, a delayed trehalose or glycogen mobilization to maintain the glycolytic flux could result in a dynamic redox state. Celton et al. reported a negative impact of aberrant NADPH homeostasis on the production of aromatic molecules [97]. In addition, a dynamic NAD^+^/NADH ratio is constantly monitored via *Sir2* in yeasts that can trigger pronounced transcriptional dynamics with possible impacts on different metabolic routes for several production scenarios [43]. Knowledge concerning dynamics of specific signaling compounds could also shed light on process performance. Alpha-ketoglutaric acid has recently been characterized as a master regulator in *E. coli*, and its role in the yield reduction in recombinant protein production was discussed by Zhang et al. [98].

Thus far, this study revealed the biological feedback of yeast cells on a specific perturbation. Follow-up work should use this finding and complementary datasets to generate models that would allow more realistic predictions of the cellular response towards industrial stimuli, with a view to enabling the *a priori* identification of biological scale-up effects.

## 4. Materials and Methods

### 4.1. Strain, Precultures and Medium

The haploid, prototrophic *Saccharomyces cerevisiae* model strain CEN.PK 113-7D [93] was used in this study and was kindly provided by Royal DSM N.V. (Delft, The Netherlands). Cells were stored at −70 °C in 1 mL aliquots supplemented with 30% (*v*/*v*) glycerol. For each experiment, yeast extract peptone dextrose (YPD) agar plates were prepared by streaking cells directly from the frozen glycerol stock and incubating for two days at 30 °C. Single colonies were picked and suspended with 5 mL YPD broth in a culture glass vial. The vials were mounted at a 45° angle on an orbital shaker and incubated for 8 h at 30 °C with 120 revolutions per minute. Subsequently, the cultures were pelleted and inoculated in shake-flask cultures with 110 mL adjusted Verduyn medium [99] and incubated over night at 30 °C on an orbital shaker with 120 revolutions per minute. To support carbon-limited growth in chemostat conditions with 22.5 g·L^−1^ glucose, the medium was designed as follows: ammonium sulfate ((NH_4_)_2_SO_4_) 15.0 g·L^−1^, monopotassium phosphate (KH_2_PO_4_) 9.0 g·L^−1^, magnesium sulfate heptahydrate (MgSO_4_·7H_2_O) 1.5 g·L^−1^, ethylenediaminetetraacetic acid ((CH₂N(CH₂CO₂H)₂)₂) 38.22 mg·L^−1^, zinc sulfate heptahydrate (ZnSO_4_·7H_2_O) 9.00 mg·L^−1^, manganese(II) chloride tetrahydrate (MnCl_2_·4H_2_O) 2.00 mg·L^−1^, cobalt(II) chloride hexahydrate (CoCl_2_·6H_2_O) 0.60 mg·L^−1^, copper(II) sulfate pentahydrate (CuSO_4_·5H_2_O) 0.60 mg·L^−1^, sodium molybdate dihydrate (NaMoO_4_·2H_2_O) 0.80 mg·L^−1^, calcium chloride dihydrate (CaCl_2_·2H_2_O) 9.00 mg·L^−1^, iron(II) sulfate heptahydrate (FeSO_4_·7H_2_O) 6.00 mg·L^−1^, boric acid (H_3_BO_3_) 2.00 mg·L^−1^, potassium iodide (KI) 0.20 mg·L^−1^, D-biotin (C_10_H_16_N_2_O_3_S) 0.10 mg·L^−1^, calcium pantothenate (C_18_H_32_CaN_2_O_10_) 2.00 mg·L^−1^, nicotinic acid (C_6_H_5_NO_2_) 2.00 mg·L^−1^, myo-inositol (C_6_H_12_O_6_) 50.00 mg·L^−1^, thiamine HCl (C_12_H_18_Cl_2_N_4_OS) 2.00 mg·L^−1^, pyridoxine HCl (C_8_H_12_ClNO_3_) 2.00 mg·L^−1^ and para-aminobenzoic acid (C_7_H_7_NO_2_) 0.40 mg·L^−1^.

### 4.2. Bioreactor and Chemostat Setup

Aerobic, carbon-limited chemostat cultivations were carried out in a 3 l stainless steel benchtop bioreactor (Bioengineering, Wald, Switzerland) with a working volume of 1.7 L. The reactor was equipped with two six-blade Rushton-type impellers, four baffles and sensors for pH (Mettler Toledo, Columbus, OH, USA), pO_2_ (PreSens, Regensburg, Germany), temperature and pressure (both Bioengineering, Wald, Switzerland). The system was operated with an overpressure of 0.3 bar, pH was controlled at 5.00 with 2 M KOH, the temperature was kept at 30 °C and aerobic conditions were maintained with bottled, ambient air supplied with 0.8 Nl·min^−1^ and bubbles were dispersed with an impeller speed of 800 rpm. Foaming was prevented throughout the process by a continuous supply of Struktol J 674 antifoam (Schill und Seilacher, Hamburg, Germany) with a pump rate of 30 μL·h^−1^. Oxygen and carbon dioxide fractions in the off-gas were logged every minute with BCP-O_2_ and BCP-CO_2_ sensors (BlueSens, Herten, Germany).

Each process was initiated as a batch fermentation by inoculating 1.6 L adjusted Verduyn medium with 0.1 L of an overnight shake-flask culture. Glucose depletion was monitored based on a sharp increase in the pO_2_ signal, which was followed by switching to chemostat conditions. The system was operated at a dilution rate of 0.1 h^−1^ with two U-120 peristaltic pumps (Watson-Marlow, Falmouth, UK). The feed pump was operated continuously at 2.83 mL·min^−1^ and the harvest pump was controlled at a higher speed relative to the feed pump via mass balancing of the bioreactor. The feed medium was continuously stirred with a magnetic stir bar to avoid gradient formation in the feed casket and the dilution rate was monitored based on the mass balance of the feed reservoir.

### 4.3. Stimulus-Response Experiment

Reference steady-state samples were drawn after five residence times with constant off-gas signals. The non-adapted response was induced by a single transition into a non-fed regime by switching off the feed pump for 2 min, followed by a continuation of the previous chemostat regime. The biological response was characterized with the below-mentioned methods for up to six hours post-stimulus. Subsequently, the feeding regime was switched to an intermittent feed. The feed pump was switched off for two minutes and switched on for seven minutes repetitively, resulting in nine-minute regime transitioning cycles. During the feed phase of every cycle, the feed rate was adjusted to 3.64 mL·min^−1^ to maintain an average dilution rate of 0.1 h^−1^. After five residence times in the intermittent feeding regime, a dynamic steady state was assumed and samples representing the adapted response were drawn. The whole chemostat process was not operated for more than 15 residence times to avoid the occurrence of laboratory evolution effects [86].

### 4.4. Sampling

The bioreactor was equipped with two custom-made, semiautomated sampling devices. For each sample port, a stainless steel broach needle (Bioengineering, Wald, Switzerland) was connected via a septum with the bioreactor and the exit was extended with a silicon tube with an inner diameter of 0.5 mm. The tube was closed with a pinch valve (model: S105, ASCO/Sirai, Bussero, Italy) to allow sampling of precise volumes enabled via time-relay-controlled valve opening (time relay model: FSM10, Tele Haase Steuergeräte, Vienna, Austria). Each sampling device was calibrated during reference steady-state conditions for each biological replicate separately and the volume deviation from the set point of five replicates for volumes between 1 and 5 mL was always below 2%. All samples were drawn after discarding the dead volume of 300 μL.

Cultivation broth samples for biomass and carbon balancing were briefly chilled on ice for degassing of the broth before distributing adequate volumes for each method.

Extracellular supernatants were obtained by directly sampling into a syringe equipped with a PES filter (Ø 30 mm, 0.22 μm pore size, ROTILABO^®^, Carl Roth, Karlsruhe, Germany) and the filtrate was collected within 5 s and stored at −70 °C.

Defined biomasses for intracellular metabolic analysis were withdrawn according to an adapted and sequential protocol employing rapid cold-methanol quenching and methanol–chloroform extraction [100]. Following procedure: 1.5 mL cultivation broth was directly injected into 10 mL methanol cooled down to −40 °C and immediately centrifuged at 5000× *g* for 5 min at −11 °C. Samples were thoroughly decanted, flash-frozen and stored at −70 °C until extraction. During high-frequency sampling periods (initial perturbation phase, up to Δt = 540 s), quenched cultivation broths were interim stored at −40 °C in a cryostat (RK20, Lauda, Lauda-Königshofen, Germany) for a maximum time of 5 min to prevent metabolite leakage [57]. The frozen cell pellets were resuspended in precooled (−20 °C) extraction buffer consisting of 50% vv^−1^ aqueous methanol solution, 100 mM ammonium acetate (pH 9.2), 2.5 mM 3-mercaptopropionic acid and 100 μM L-norvaline as internal standard (extraction). Added volumes were adjusted to achieve constant biomass concentrations (8.5 g·L^−1^) and the sample temperature was kept below −20 °C by rotational mixing (Δt = 30 s) and chilling in a cryostat (−40 °C) during complete resuspension. Next, the same volume of precooled (−20 °C) chloroform was added and the mixed suspension was incubated for 2 h at −20 °C and 1 h at room temperature in a rotary overhead shaker. Afterwards, the samples were centrifuged at 20,000× *g* for 10 min at 4 °C and the upper aqueous methanol phase containing polar metabolites was carefully removed and stored at −70 °C until measurement.

### 4.5. Off-Gas Deconvolution

A prerequisite for proper off-gas analysis in stimulus-response experiments is a suitable approach for signal deconvolution. Long tubing lines and foam traps between fermenter and sensors led to the formation of mixing chambers, causing a sensor delay of several minutes and increased apparent time constants versus the reported 55 s for BCP-O_2_ and BCP-CO_2_ sensors [101]. Step experiments were carried out under experimental conditions with H_2_O as a broth substituent to identify delay times and time constants for each sensor. Correction of the O_2_ and CO_2_ signals during the stimulus-response experiments was computed based on the methodology by Theobald et al. [102]. For a complete description of the step experiments and mathematical deconvolution approach, the reader is referred to Appendix A.

### 4.6. Dry Matter of Biomass Determination

Triplicated 5 mL volumes were vacuum-filtered through dried and tared PES membrane disc filters (Ø 47 mm, Type 154, Sartorius, Göttingen, Germany). Filters were, subsequently, washed with 15 mL demineralized water and dried at 70 °C until mass constancy was observed. Finally, filters with biomass cakes were brought to room temperature in a desiccator and were weighed again. The calculated weight of the biomass cake was normalized to the sample volume and expressed as dry matter of biomass (*DMB*).

### 4.7. Extracellular Metabolite Quantification

Frozen supernatant samples were thawed on ice and glucose was measured using a UV-based enzyme test kit (art. no. 10716251035, r-biopharm AG, Darmstadt, Germany). The free ammonium concentration was quantified with the LCK302 cuvette test kit (Hach Lange, Düsseldorf, Germany). Each kit was performed according to the manufacturer’s instructions on a spectrophotometer (Hach Lange, Düsseldorf, Germany). Unknown extracellular carbon was calculated based on an organic carbon balance of broth supernatant using a total carbon analyzer (Multi N/C 2100s, AnalytikJena, Jena, Germany).

### 4.8. Determination of Intracellular Carbohydrate Storage Pools

Intracellular glycogen and trehalose levels were determined based on the protocols reported by Parrou et al. and Suarez-Mendez et al. [103,104]. Frozen pellets were resuspended in 250 μL 0.25 M sodium carbonate and incubated for 3 h at 95 °C. Subsequently, the pH was adjusted to 5.5 by addition of 150 μL^−1^ M acetic acid and 600 μL 0.2 M sodium acetate (pH 5.2, adjusted with acetic acid). The sample was split into a 480 μL and a 466 μL aliquot. The first was treated with 20 μL of α-amyloglucosidase (~70 U/mL, catalog number: 10115, Merck, Darmstadt, Germany) at 57 °C overnight to determine glycogen expressed as liberated glucose equivalents. For trehalase determination, the pH of the 466 μL aliquot was adjusted slightly upwards by the addition of 30 μL of 0.2 M sodium acetate, and trehalose was hydrolyzed to glucose by the addition of 4 μL trehalase (2.27 U/mL, catalog number: T8778, Merck, Darmstadt, Germany) and incubated at 37 °C overnight. Glucose equivalents were measured with the UV-based enzyme test kit (art. no. 10716251035, r-biopharm AG, Darmstadt, Germany).

### 4.9. Determination of Intracellular Metabolites Measured via LC-MS/MS

Quantitative metabolome analyses of intracellular *S. cerevisiae* extracts (see Section 4.4) were conducted on an Agilent 1200 HPLC system coupled with an Agilent 6410B triple-quadrupole (QQQ) mass spectrometer with a classical electrospray ionization (ESI) interface.

Analytical preparation of sample extracts and chromatographic separation of nonderivatized polar metabolites by alkaline polymer-based zwitterionic hydrophilic interaction chromatography (ZIC-pHILIC) were performed as previously described [105,106]. Defined standard mixtures and samples with adapted dilution containing 50 μM 2-keto-3-deoxy-6-phosphogluconate (KDPG) and α-amino isobutyric acid (AIBA) as global internal standard (measurement) were injected (5 μL) into a Sequant ZIC-pHILIC column (150 × 2.1 mm, 5 μm, Merck Millipore, Darmstadt, Germany) equipped with a guard column (20 × 2.1 mm, 5 μm, Merck Millipore, Darmstadt, Germany) maintained at 40 °C.

Analogue measurements of previously derivatized (Phenylhydrazine) α-keto acids (αKG, PYR, GXY) were performed by an adapted LC-MS/MS protocol [107] using 50 μM α ketovalerate as internal standard (derivatization/measurement). Derivatized analytes were separated under acidic conditions (pH 3.0) by reverse-phase liquid chromatography (RPLC) [108]. Samples were injected (5 μL) onto a ZORBAX SB-C18 column (150 × 4.6 mm, 5 μm, Agilent Technologies, Waldbronn, Germany) with a guard column (12.5 × 4.6 mm, 5 μm, Agilent Technologies, Waldbronn, Germany) maintained at 40 °C.

Targeted metabolites were detected with high selectivity in multiple reaction monitoring (MRM) mode using established and preoptimized precursor-to-product transitions and MS/MS parameters with a mass resolution of 0.1 u. Intracellular metabolite pools were absolutely quantified by a threefold standard addition of defined amounts of reference standard mixes (internal calibration). Applied amounts were adjusted according to previously estimated concentration levels and linear dynamic ranges of the targeted metabolites [109]. The absolute concentration levels of the AxP species were normalized to results from a reference method [54] to compensate for known HILIC-specific peak tailing effects in iron-based LC systems [110]. The normalization factors (ATP: 2.71, ADP: 1.88 and AMP: 1.21) were calculated from analogues steady-state RS samples and were applied conformably.

### 4.10. Characterization of the Endometabolome Relaxation Pattern

A classical, metric multidimensional scaling approach [111] was chosen to quantify and visualize dissimilarities between the different time points. Concentrations of all 29 intracellular metabolites, except pyruvic acid, were considered and min–max normalized. In the next step, the Euclidean distance matrix was computed with the function *dist* and used as an input for *cmdscale*, which was limited to a two-dimensional representation of the sample distances (k = 2). All computations were executed in the R environment (version 1.4.1106) with the package *stats* (version 4.1.0).

### 4.11. Total Carbon and Nitrogen Determination

One milliliter of fermentation broth was mixed with nine milliliters of 36.84 mM KOH to prevent loss of inorganic carbon in the form of dissolved carbonate. Next, the 1:10 diluted sample was measured in octuplicate, and undiluted supernatant (also stabilized with 36.84 mM KOH) was measured in quadruplicate with a multi N/C 2100 S composition analyzer (Analytik Jena, Jena, Germany). The system was calibrated according to the method of Buchholz et al. [112]. Nitrogen concentrations were directly measured and organic carbon was determined based on the difference between the total carbon and the inorganic carbon fractions.

## 5. Conclusions

This study set out to investigate the response of *Saccharomyces cerevisiae* during industrially relevant transitions between carbon limitation and starvation, and vice versa. The intracellular metabolite analysis provided a solid dataset for future modeling efforts and revealed distinct phenomena that helped to explain biological scale-up effects. The experimental design allowed the observation of several dynamics from the allosteric control of specific intermediates to global phenotypic changes as a response to the applied substrate gradient. In particular, a distinct mode was uncovered where yeasts sacrificed catabolic reduction power to sustain ongoing anabolic demands under acute carbon starvation conditions. A natural progression of this work is to expand the obtained knowledge by analyzing gene expression dynamics to investigate (i) if and how metabolic stimuli are propagated in cells exposed to an industrially relevant famine perturbation and (ii) to use the obtained data for setting up data-driven models for a rational scale-up/scale-down.

## Figures and Tables

**Table 1:** Process balances at sample points relevant for this study.

Sample Point	Carbon Recovery (% ± s.d.)	Nitrogen Recovery (% ± s.d.)	Available Electron Recovery (% ± s.d.)
Steady-state RS	98.8 ± 0.7	102.5 ± 6.5	97.5 ± 0.7
30 min post-stimulus	102.2 ± 1.2	102.8 ± 6.6	100.2 ± 1.2
60 min post-stimulus	97.2 ± 0.6	99.0 ± 3.5	96.4 ± 0.6
120 min post-stimulus	98.6 ± 0.9	98.9 ± 3.5	97.4 ± 0.9
180 min post-stimulus	98.3 ± 0.8	98.9 ± 3.6	97.2 ± 1.0
240 min post-stimulus	98.4 ± 0.5	98.9 ± 3.6	97.3 ± 0.5
360 min post-stimulus	99.0 ± 0.6	101.2 ± 4.6	97.8 ± 0.7
Steady-state DS	100.7 ± 0.7	101.0 ± 7.8	99.1 ± 1.0

All percentages express means ± standard deviation (s.d.) of three biological replicates. RS, reference steady state; DS, dynamic steady state.

**Table 2:** Yeast kinetics at the steady states RS (reference) and DS (after dynamic perturbation).

Parameter	Dimension	Steady-State RS	Steady-State DS	Change (%)	Welch Test (*p*-Value)
*D*	h^−1^	0.101 ± 0.001	0.100 ± 0.002	n.s.	>0.05
*Y_DMB_*_/glucose_	g*_DMB_*·g_glucose_^−1^	0.494 ± 0.005	0.498 ± 0.002	n.s.	>0.05
−*q*_glucose_	mmol·g*_DMB_*^−1^·h^−1^	1.13 ± 0.01	1.12 ± 0.02 *	n.s.	>0.05
−*q*_oxygen_	mmol·g*_DMB_*^−1^·h^−1^	2.52 ± 0.01	2.63 ± 0.04 *	+4.3	0.03
*q*_carbon dioxide_	mmol·g*_DMB_*^−1^·h^−1^	2.71 ± 0.02	2.83 ± 0.04 *	+4.3	0.02
*Y*_oxygen/glucose_	mol·mol^−1^	2.23 ± 0.03	2.34 ± 0.03 *	+4.9	0.02
*−q*_ammonia_	mmol·g*_DMB_*^−1^·h^−1^	0.86 ± 0.04	0.94 ± 0.07 *	n.s.	>0.05
*q*_other carbon_	mmol_C_·g*_DMB_*^−1^·h^−1^	0.140 ± 0.008	0.121 ± 0.003	−13.4	0.04

All values represent means ± standard deviation of three biological replicates. Values marked with an asterisk indicate an averaged parameter over one 9 min perturbation cycle. *DMB*, dry matter of biomass; RS, reference steady state; DS, dynamic steady state; n.s., not significant.

**Figure A1:**
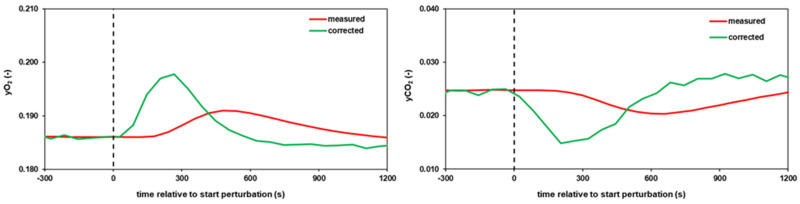
Exemplary deconvolution results for O_2_ and CO_2_ signals of one replicate after a single perturbation (see Figure 3c). Deconvolution of O_2_ (left panel) and CO_2_ (right panel) signals (green) was calculated based on equations (A1) and (A2), parameters from Table A1 and plotted against raw signals (red).

## Data Availability

The data that support the findings of this study are available from https://dataverse.nl/dataverse/minden-metabolites (last accessed: 17 March 2022).

## Appendix A. Off-Gas Deconvolution

A prerequisite for the off-gas analysis in stimulus-response experiments is a suitable approach for data deconvolution. Long tubing lines and foam traps between fermenter and sensors led to the formation of mixing chambers, causing a sensor delay of several minutes and increased apparent time constants versus the reported 40–55 s for BCP-O_2_ and BCB-CO_2_ sensors [101]. This could cause misguided readouts such as strong RQ dynamics that might not at all be caused by biological effects. To compensate for this system characteristic, step experiments under experimental conditions were carried out to acquire delay times and time constants for the whole “fermenter → sensor” unit, as laid out in Table A1. A correction of measured off-gas signals was calculated according to the procedure of Theobald et al. [102]:

(A1) $${\left(Y_{O_{2}}^{\omega }\right)}_{F}\left(t-\tau_{d}^{O_{2}}\right)={\left(Y_{O_{2}}^{\omega }\right)}_{A}+\tau_{1}^{O_{2}}\frac{d{\left(Y_{O_{2}}^{\omega }\right)}_{A}}{dt}$$

(A2) $${\left(Y_{CO_{2}}^{\omega }\right)}_{F}\left(t-\tau_{d}^{CO_{2}}\right)={\left(Y_{CO_{2}}^{\omega }\right)}_{A}+\tau_{1}^{CO_{2}}\frac{d{\left(Y_{CO_{2}}^{\omega }\right)}_{A}}{dt}$$

with

${\left(Y_{O_{2}}^{\omega }\right)}_{F}$ molar fraction of O_2_ in the fermenter broth;

${\left(Y_{CO_{2}}^{\omega }\right)}_{F}$ molar fraction of CO_2_ in the fermenter broth;

${\left(Y_{O_{2}}^{\omega }\right)}_{A}$ measured molar fraction of O_2_ at the sensor;

${\left(Y_{CO_{2}}^{\omega }\right)}_{A}$ measured molar fraction of CO_2_ at the sensor;

t time of data logging for O_2_ and CO_2;_

$\tau_{d}^{O_{2}}$ delay time of O_2_ signal;

$\tau_{d}^{CO_{2}}$ delay time of CO_2_ signal;

$\tau_{1}^{O_{2}}$ time constant of BCP-O_2_ sensor and gas line “fermenter → sensor”;

$\tau_{1}^{CO_{2}}$ time constant of BCP-CO_2_ sensor and gas line “fermenter → sensor”.

**Table A1:** Parameters for off-gas deconvolution. Delay times (τ_d_) and time constants (τ_1_) were derived from step experiments, where the reactor system was operated with water, but otherwise equal to fermentation conditions. Each parameter was derived from two-step experiments, where aeration was switched from a calibration gas mixture (2.00% O_2_, 8.99% CO_2_) to ambient air (20.94% O_2_, 0.04% CO_2_).

Analyte	Sensor	Manufacturer	$\tau_{d}\;(s)$	$\tau_{1}\;(s)$
Oxygen	BCP-O_2_	BlueSens, Herten, Germany	92	381
Carbon dioxide	BCP-CO_2_	BlueSens, Herten, Germany	155	490

Figure A1 shows an exemplary correction for both, oxygen and carbon dioxide signals against raw signal readouts after a single perturbation (Figure 3c), as indicated by the dashed lines. It became obvious that this procedure was essential to compensate for delays and curve flattening due to a total of 3.2 L mixing volume in the off-gas line.

## Appendix B. Steady-State Amino Acid Concentrations

A total of 18 amino acids was also monitored during the SRE experiment under both conditions. However, due to the absence of significant dynamics during the perturbation, only steady-state values were reported in Table A2.

**Table A2:** Amino acid concentrations during reference and dynamic steady-state conditions. For the dynamic steady state, average pool concentrations over one perturbation cycle were reported.

Amino Acid	Steady-State RS (μmol·g*_DMB_*^−1^)	Steady-State DS (μmol·g*_DMB_*^−1^)	Change (%)	Welch Test (*p*-Value)
glycine	2.37 ± 0.02	1.05 ± 0.28	−56	7.4 × 10^−5^
L-methionine	0.18 ± 0.04	0.05 ± 0.01	−72	2.9 × 10^−2^
L-serine	3.29 ± 0.19	1.29 ± 0.51	−61	6.9 × 10^−5^
L-proline	6.15 ± 1.12	2.17 ± 1.08	−65	7.4 × 10^−3^
L-threonine	10.5 ± 4.5	9.8 ± 2.9	−7	n.s.
L-glutamine	149 ± 10	103 ± 20	−31	2.6 × 10^−3^
L-asparagine	8.64 ± 1.31	8.7 ± 1.60	+1	n.s.
L-glutamic acid	449 ± 26	390 ± 71	−13	n.s.
L-aspartic acid	22.9 ± 5.7	22.4 ± 5.8	−2	n.s.
L-lysine	3.79 ± 0.47	4.19 ± 0.12	+11	n.s.
L-arginine	17.8 ± 0.3	9.8 ± 0.9	−45	5.9 × 10^−7^
L-tyrosine	1.92 ± 0.05	0.41 ± 0.05	−78	1.5 × 10^−3^
L-tryptophane	0.36 ± 0.11	0.11 ± 0.01	−68	n.s.
L-phenylalanine	0.85 ± 0.09	0.28± 0.05	−66	4.1 × 10^−3^
L-valine	22.1 ± 2.6	12.6 ± 4.1	−43	5.0 × 10^−3^
L-leucine	0.69 ± 0.09	0.38 ± 0.1	−46	6.8 × 10^−3^
L-isoleucine	1.45 ± 0.02	0.79 ± 0.19	−46	3.1 × 10^−4^
L-alanine	84.9 ± 8.0	43.4 ± 16.7	−49	1.5 × 10^−3^

All values represent means ± standard deviation (s.d.) of three biological replicates. n.s., not significant.
